# Perampanel Confirms to Be Effective and Well-Tolerated as an Add-On Treatment in Patients With Brain Tumor-Related Epilepsy (PERADET Study)

**DOI:** 10.3389/fneur.2020.00592

**Published:** 2020-06-25

**Authors:** Antonietta Coppola, Alessia Zarabla, Andrea Maialetti, Veronica Villani, Tatiana Koudriavtseva, Emilio Russo, Agostino Nozzolillo, Chiara Sueri, Vincenzo Belcastro, Simona Balestrini, Edoardo Ferlazzo, Diana Giannarelli, Leonilda Bilo, Marta Maschio

**Affiliations:** ^1^Department of Neurosciences, Reproductive and Odontostomatological Sciences, Federico II University, Naples, Italy; ^2^Center for Tumor-Related Epilepsy, UOSD Neuroncology, IRCCS IFO Regina Elena National Cancer Institute, Rome, Italy; ^3^Department of Science of Health, Magna Græcia University of Catanzaro, Catanzaro, Italy; ^4^Department of Medical and Surgical Sciences, Magna Graecia University, Catanzaro, Italy; ^5^Regional Epilepsy Center, Great Metropolitan Hospital “Bianchi-Melacrino-Morelli”, Reggio Calabria, Italy; ^6^Child Neuropsychiatry Unit, Department of Mental Health, ASST-LARIANA, Como, Italy; ^7^Department of Clinical and Experimental Epilepsy, UCL Queen Square Institute of Neurology and Chalfont Center for Epilepsy, Bucks, United Kingdom; ^8^Biostatistic Unit, IRCCS IFO Regina Elena National Cancer Institute, Rome, Italy

**Keywords:** brain tumor-related epilepsy, perampanel, efficacy, tolerability, quality of life

## Abstract

**Background:** Epilepsy is one of the most common symptoms of brain tumors. It is often drug resistant and generally worsen patients' quality of life (QoL). Brain tumors release glutamate among other mediators, contributing to seizures onset, and this is accompanied by an increased AMPA receptors' expression on neuronal cells' membrane. Perampanel (PER) is a relatively new antiseizure medication (ASM) that acts as a selective non-competitive AMPA receptors' antagonist. Given its mechanism of action, we aimed to evaluate through a prospective, observational study, the efficacy and safety of PER as an add-on treatment in patients with brain tumor-related epilepsy (BTRE). The study was called PERADET.

**Methods:** Thirty-six adult patients (intention to treat population-ITT) affected by BTRE, with uncontrolled focal-onset seizures treated with 1–3 ASMs were recruited from four Italian epilepsy centers. Perampanel was added-on, titrated from 2 mg/day up to a maximum of 12 mg/day. Tumor history and therapy, type, and seizures frequency, previous ASMs were collected at 6 and 12 months. A battery of QoL tests were administered at baseline, 6 and 12 months. The primary endpoint was to assess the efficacy of PER by calculating the percent change in seizure frequency and the responder rate. The secondary endpoints were tolerability, retention rate at 12 months, and improvement in quality of life.

**Results:** At the end of 12 months, 21 patients (per protocol population-PP) were available for evaluation. In this population the responder rate (percentage of patients who experienced a 50% or greater reduction in seizure frequency) was 90.4 with 33.3% of patients being seizure-free. In the ITT group the responder rate at the end of 12 months was 66.6 with 25% of patients being seizure free. PER was well tolerated (30.6% of patients experienced an adverse event, none was severe; three needed a treatment interruptions).

**Conclusions:** Our study indicate that PER may be efficacious against BTRE as suggested by its mechanism of action and our current knowledge on mechanisms of brain tumor epileptogenicity.

**Trial Registration Number (TRN):** (Prot. n° 0008872.25-06-2019); RS 919/17.

## Background

Epilepsy is one of the most common (up to 70%) symptoms of brain tumors (1). About 30–50% of patients present with seizures as the first symptoms and 10–30% will develop seizures later during the disease course (1). In these conditions, epilepsy is often drug resistant and about 40% of patients can be forced to take polytherapy that can contribute to the burden of living with a brain tumor also negatively influencing their quality of life (QoL) (1, 2).

Pharmacotherapy of brain tumor-related epilepsy (BTRE) is complicated by possible loss of efficacy over time and potential interactions between anti-seizure medications (ASMs) and anticancer therapies, which may expose patients to an increased risk of adverse effects (AEs). Within this context, the second-generation ASMs are generally preferred over older-generation, in order to minimize the risks of interactions (3).

The widely held theory underlying BTRE is a peri-tumoral neuronal hyperexcitability. This can be due to weakened GABAergic inhibitory function and/or paradoxical GABA mediated excitation and to excessive glutamatergic excitatory activity. The latter is due to increased glutamate release (4, 5), reduced glutamate uptake within tumor and reduced clearance via non-tumor cells (6), altered glutamate receptor expression (7–9). AMPA receptor seems to be the most expressed subtype in different brain tumors (7–9). Thus, Perampanel (PER), a relatively new ASM acting as a selective non-competitive AMPA antagonist, seems to be a rational drug choice in BTRE (10). This drug has been initially licensed as adjunctive treatment for patients with focal and focal to bilateral seizures and more recently for generalized onset seizures and as a monotherapy in some countries (11). Subsequently, two case studies on BTRE have been published, and although anecdotally, these results were encouraging (12–16). In our previous retrospective study on 11 subjects followed for 12 months, we found a high rate of responders (81.8%) and five patients become seizure free, suggesting that PER could be a therapeutic option in BTRE in agreement with its supported neurophysiopathological rational choice (13).

Herein, we report the results of the PERADET study, the first prospective, multicenter observational study evaluating, over a period of 12 months, the efficacy, tolerability, and impact on QoL of PER as an add-on treatment in BTRE patients with uncontrolled seizures.

## Methods

Patients fulfilling the inclusion criteria were consecutively recruited in four Italian Epilepsy Centers (Naples, Rome, Como, and Reggio Calabria) over a period of 12 months. Patients older than 18 years old and affected by BTRE with uncontrolled seizures were included in the present study if they had at least three focal-onset seizures during the 6 weeks baseline period (as indicated in the approved protocol), were treated with at least one appropriately chosen ASM, had a brain MRI performed in the last 3 months preceding the recruitment, and were able to sign the informed consent. Only primary brain tumors were included in the study. Pregnancy or planned pregnancy, surgery in the 2 months preceding the Evaluation Visit or planned surgery, moderate or severe renal and/or liver failure, hematological active diseases, non-epileptic seizures, or history of drug or alcohol abuse were exclusion criteria.

After written informed consent was obtained, PER was added-on starting from 2 mg/day for 2 weeks. Then PER was titrated with increments of 2 mg every 4 weeks up to the desired dose or a maximum of 12 mg/day depending on clinical outcome.

The primary endpoint was to assess the efficacy of PER by calculating the percent change in seizure frequency (mean number of seizures/month at 12 months—mean number of seizures/month at baseline), the responder rate (percentage of patients who experienced a 50% or greater reduction in seizure frequency) and seizure freedom between baseline and last follow up (12 months).

The secondary endpoints were tolerability, retention rate at 12 months, evaluation of quality of life modification and analysis, and comparison of subgroups obtained by patients' stratification by oncological disease related factors. Safety was monitored by assessing: the number and percentage of patients having any adverse event; the number and percentage of patients having any drug-related adverse event recorded as Grade 3 or 4 or as Serious Adverse Event (according to the CTCAE-Common Terminology Criteria for Adverse Events of the US National Cancer Institute); any change from baseline in vital signs and laboratory results (hematology and blood chemistry).

Quality of life questionnaire for epilepsy (QOLIE 31-p version2 version 1.0) was obtained at baseline and at 12 months. Becks hopelessness scale (BHS) and Aggression questionnaire (AQ) were obtained for a subgroup of patients at baseline, 6 and 12 months. Seizure frequency, concomitant antiseizure medication (ASM), chemotherapy and radiotherapy data, brain MRI, EEG, histology, and molecular data (i.e., isocitrate dehydrogenase 1-IDH1 and O6-Methylguanine-DNA-methyltransferase-MGMT expression) were collected and analyzed.

### Statistical Analysis

Based on a previous study of a large population with drug-resistant epilepsy (17), we estimated an average seizure frequency of 11 per month prior the introduction of perampanel with a standard deviation (SD) of 10.

A sample size of 36 was projected to provide 80% power at a significance level of 5% and an expected effect size of about 50%.

In this study, qualitative items were summarized by absolute counts and percentages while quantitative variables were reported as means and standard deviations or median and range. Paired Student's *t*-test was used for assessing differences between the two evaluations (baseline and follow-up). A *P* < 0.05 was considered as statistical significant and IBM-SPSS ver. 21.0 software was used for analysis.

## Results

We recruited 36 patients (Intention to treat population-ITT), 13 females (36.1%) and 23 males (63.9%) with a median age of 46 years (range 15–75) and a mean number of seizures prior to study entry of 9.1 ± 12.8 (SD) per month. Fourteen patients (38.9%) had focal seizures with preserved awareness, seven (19.4%) had focal onset seizures with loss of awareness, 11 (30.6%) had focal to bilateral, and four (11.1%) had apparently generalized seizures (with a clear focal focus at EEG). Nineteen patients (52.8%) were on monotherapy (the most used drug on monotherapy was levetiracetam in 12 patients) and 17 (47.2%) were on polytherapy (three were under three ASMs and 12 were on bitherapy; the most used regimen was lacosamide plus levetiracetam in five individuals) at the time of evaluation. The concomitant ASMs treatment were not changed during this study. Benzodiazepines taken occasionally were not considered among the ASMs regimen. Table 1A summarizes the characteristics of our population.

**Table 1A:** Characteristics of our population.

Total number of recruited patients	36
**SEX**	
Male	23 (63.9%)
Female	13 (36.1%)
Age in years median (range)	46 (15–75)
**TYPE OF SEIZURE**	
Focal with preserved awareness	14 (38.9%)
Focal with compromised awareness	7 (19.4%)
Focal to bilateral	11 (30.6%)
(Apparently) Generalized	4 (11.1%)
**NUMBER OF SEIZURES at baseline** (mean ± SD)	9.1 ± 12.8
**CONCOMITANT ASMs TREATMENT**	
Monotherapy	19 (52.8%)
	12 LEV
	2 PB
	2 OXC
	1 LTG
	1 PHT
	1 CBZ
Polytherapy	17 (47.2%)
	1 VPA + LEV + LTG
	1 OXC + LEV + LCM
	1 CBZ + LEV + CLN
	5 LCM + LEV
	1 ZNS + OXC
	1 VPA + LEV
	1 VPA + LCM
	1 VPA + CBZ
	1 CBZ + LCM
	1 OXC + LCM

*ASMs, anti-seizure medications; CBZ, carbamazepine; CLN, clonazepam; LCM, lacosamide; LEV, levetiracetam; LTG, lamotrigine; OXC, oxcarbazepine; PB, phenobarbital; PHT, phenytoin; VPA, valproate; ZNS, zonisamide*.

Table 1B indicates the histology of the tumors, grade, site, and related treatment including surgery, chemotherapy, and radiotherapy. Eleven patients (30.6%) had a low grade glioma (LGG), 14 (38.9%) had a high grade glioma (HGG), seven (19.4%) had a glioblastoma and four (11.1%) had an unclassified tumor. Six patients (16.7%) were IDH1 mutated; 10 (27,8%) were negative; 20 (55.6%) were unknown. Seven patients (19.4%) were MGMT mutated; four (11.1%) were unmetilated; in 25 (69.5%) the methylation status was not known).

**Table 1B:** Characteristics of the tumor and treatment.

**HISTOLOGY**	
Low grade glioma	11 (30.6%)
High grade glioma	14 (38.9%)
Glioblastoma	7 (19.4%)
Other	4 (11.1%)
**GRADE**	
Low	12 (33.3%)
High	21 (58.3%)
Other	3 (8.3%)
**TUMOR SITE**	
Frontal	12 (33.3%)
Temporal	6 (16.7%)
Parietal	3 (8.3%)
Multilobular	14(38.9%)
Occipital	1 (2.8%)
**SURGERY**	
UKN	2 (5.6%)
Biopsy	3 (8.3%)
Gross total resection (>90%)	13 (36.1%)
Partial resection (<90%)	18 (50.0%)
**CHEMOTHERAPY**	
Yes, before PER treatment	12 (33.3%)
Yes, before and during PER treatment	15 (41.7%)
Yes, during PER treatment	2 (5.6%)
No	7 (19.4%)
**CHEMOTHERAPY**	
No	7 (19.4%)
Temozolomide	16 (44.4%)
Fotemustine	4 (11.1%)
Bevacizumab	1 (2.8%)
Other	5 (13.9%)
Unknown	3 (8.3%)
**RADIOTHERAPY**	
Yes, before PER treatment	12 (33.3%)
Yes, during PER treatment	3 (8.3%)
No	19 (52.8%)
Unknown	2 (5.6%)
**IDH1 MUTATION**	
Mutated	6 (16.7%)
Non-mutated	10 (27.8%)
Unknown	20 (55.6%)
**MGMT METHYLATION STATUS**	
Methylated	7 (19.4%)
Unmethylated	4 (11.1%)
Unknown	25 (69.5%)

At the last follow up, one patient (2.8%) was treated with 2 mg/day of PER; seven (19.4%) with 4 mg/day; 14 (38.9%) with 6 mg/day; one (2.8%) with 7 mg/day; nine (25%) with 8 mg/day; three (8.3%) with 10 mg/day and one patient with 12 mg/day (2.8%). The final dose used was established according to clinical response and/or intolerable side effects.

At the end of 12 months, 21 patients were available for evaluation (PP-per protocol population). The mean seizure frequency was significantly reduced for both the ITT population [from 9.1 ± 12.8 to 2.6 ± 5.0 seizure per month (*p* = 0.007)] and the PP population [from 10.7 ± 14.7 to 1.7 ± 4.2 seizure per month (*p* = 0.002)]. We also evaluated the efficacy with regard to seizures' type (focal aware/unaware seizures and focal to bilateral/apparently generalized seizures) in the ITT population and we observed a statistically significant difference both in patients with focal aware/unaware seizures (10.7 ± 14.9 vs. 3.7 ± 6.3; *p* = 0.026) than in those with focal to bilateral/apparently generalized seizures (pre: 6.7 ± 9.1 vs. post 1.0 ± 1.2; *p* = 0.028).

Responder rate at 12 months in 21 patients was 90.4%: seven patients were seizure-free (33.3%), 12 had a seizure reduction ≥50% (57.1%), one remained stable and one had a reduction ≤50%. Responder rate at the last follow-up available in the whole population (36 patients) was 66.6%: nine patients were seizure-free (25%), 16 had a seizure reduction ≥50% (41.6%), five remained stable, five have a reduction ≤50% and two worsened (see Table 2A).

**Table 2A:** Results. Primary outcomes: seizure reduction, responder rate.

**PERAMPANEL (mg/die)**		**Number (%)**	
2		1 (2.8)	
4		7 (19.4)	
6		14 (38.9)	
7		1 (2.8)	
8		9 (25.0)	
10		3 (8.3)	
12		1 (2.8)	
**Seizures outcome**	**Mean number of seizures pre**	**Mean number of seizures post**	***P*****-value**
**ITT** (36 pts)	9.1 ± 12.8	2.6 ± 5.0	0.007
**PP** (only evaluable at 12 months) (21 pts)	10.7 ± 14.7	1.7 ± 4.2	0.002
**Seizure outcome (details)**	**Number of patients**	**Percentage**	
**ITT** (36 pts)	9 seizure-free	25%	
	15 seizure reduction	41.6%	
	≥50%		
	5 stable	13.8%	
	5 reduction ≤ 50%	13.8%	
	2 worsened	5.5%	
**Responder rate (ITT)** (seizure free + ≥50% seizure reduction)	24/36	**66.6%**	
**PP** (Only evaluable at 12 months) (21 pts)	7 seizure-free	**33.3%**	
	12 seizure reduction	57.1%	
	≥50%		
	1 stable	4.7%	
	1 reduction ≤ 50%	4.7%	
**Responder rate/total (PP)** (seizure free + ≥50% seizure reduction)	19/21	**90.4%**	
**Seizures outcome (grade) ITT**	**Mean number of seizures pre**	**Mean number of seizures post**	***P*****-value**
Low grade	10.4 ± 16.8	1.4 ± 2.3	0.10
High grade	7.6 ± 10.5	3.4 ± 6.2	0.01
**Seizures outcome (type of seizure) ITT**	**Mean number of seizures pre**	**Mean number of seizures post**	***P*****-value**
Focal	10.7 ± 14.9	3.7 ± 6.3	0.026
Bilateral/app. generalized	6.7 ± 9.1	1.0 ± 1.2	0.028

*The bold has been used to emphasize important results*.

We evaluated the differences in mean seizure number change in LGG and HGG patients in the ITT population. There were not statistically significant differences in LGG patients (mean seizure number pre PER treatment: 10.4 ± 16.8 vs. mean seizure number post PER treatment:1.4 ± 2.3; *p* = 0.10). Conversely, we observed a statistically significant difference in HGG patients (mean seizure number pre: 7.6 ± 10.5 vs. mean seizure number post: 3.4 ± 6.2; *p* = 0.01; see Table 2A).

**Figure 1:**
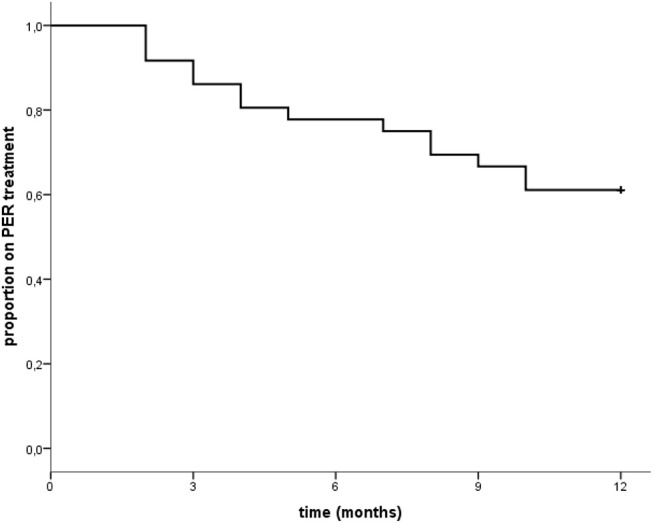
“Time to treatment withdrawal from Perampanel: at the end of 12 months 22 patients out of 36 (61.1% as shown in the graph) were still on PER treatment. However, one patient was excluded from the final retention rate calculation as he early terminated the study due to disease progression. Thus, final retention rate was 58.3% (21/36 patients).

Eleven patients (11/36; 30.6%) reported adverse events. None of these was severe. Two experienced anxiety, two irritability/aggressiveness, five dizziness, and two fatigue/tiredness. Only three of them discontinued the medication (two because of dizziness and another because of aggressiveness), while two ameliorated after dose reduction. The remaining six ameliorated spontaneously (See Table 2B). These did not seem to correlate with the dosage regimen indeed seven out of 11 experienced the adverse event at low dosage (4–6 mg/day) (see Supplementary Tables 1, 2). Eleven patients (30.6%) manifested a progression of the oncological disease during this study, and seven of them dropped out. Other drop outs were due to non-adherence to treatment (two patients), adverse events (three patients), death (two patients), worsening of seizures (one patient). Retention rate at 12 months was 58.3% (21/36 patients were still on PER at the end of the study; one patient retained PER although he early terminated his participation at the present study because of disease progression) (see Table 2B and Figure 1).

**Table 2B:** Results Secondary outcome: adverse events.

**ADVERSE EVENTS (AEs)**	
Yes	11 (30.6%)
No	25 (69.4%)
**TYPE OF AEs**	
Anxiety	2
Aggressiveness	2
Dizziness	5
Fatigue	2
**MEASURE TAKEN**	
None	6
Dose reduction	2
Treatment interruption	3
**DISEASE PROGRESSION**	
Yes	11 (30.6%)
No	25 (69.4%)
**DROP-OUTS**	
Low compliance	2
Disease progression	7
Adverse events	3
Worsening	1
Death	2
**Retention rate**	21/36 pts (58.3%)

The IDH1 mutated condition seemed to affect positively the seizures frequency outcome (see Table 2C). Indeed IDH1 mutated patients in the ITT population obtained a mean number of seizure reduction from 11.4 ± 12.3 to 5.9 ± 8.8 (*p* = 0.02) while IDH non mutated patients decreased from 11.0 ± 19.3 to 1.0 ± 1.2 (*p* = 0.13). This reduction is not significant due to high variability in total seizures' number. The MGMT methylated patients seemed to better respond to PER treatment with significant seizures reduction (*p* = 0.04 for both ITT and PP populations). Regarding disease progression, both groups (11 patients with tumor progression and 25 patients without tumor progression during follow-up) had a significative seizure reduction between baseline and final follow-up. In addition patients without a disease progression had a more significant seizure reduction compared to patients with a disease progression (*p* = 0.01 for both the ITT for the PP population; see Table 2C).

**Table 2C:** Results: outcome with regard to IDH1 mutated condition and MGMT methylated condition.

**IDH1 mutated (6 pts)**	**Mean number of seizures pre-treatment**	**Mean number of seizures post-treatment**	***P*-value**
**ITT** (6 pts)	11.4 ± 12.3	5.9 ± 8.8	0.02
PP Only evaluable at 12 months (4 pts)	9.8 ± 13.5	5.4 ± 9.7	0.11
**IDH1 non-mutated (10 patients)**	**Mean number of seizures pre-treatment**	**Mean number of seizures post-treatment**	***P*****-value**
**ITT** (10 pts)	11.0 ± 19.3	1.0 ± 1.2	0.13
PP Only evaluable at 12 months (5 pts)	20.8 ± 24.5	0.2 ± 0.2	0.15
**MGMT methylated (7 pts)**	**Mean number of seizures pre-treatment**	**Mean number of seizures post-treatment**	***P*****-value**
**ITT** (7 pts)	14.3 ± 13.1	3.4 ± 7.4	0.04
PP Only evaluable at 12 months (6 pts)	16.3 ± 13.0	3.6 ± 8.0	0.04
**MGMT non-methylated (4 pts)**	**Mean number of seizures pre-treatment**	**Mean number of seizures post-treatment**	***P*****-value**
**ITT** (4 pts)	16.7 ± 28.9	1.0 ± 1.4	0.36
PP Only evaluable at 12 months (2 pts)	32.5 ± 38.9	0.5 ± 0.7	0.46
**Pts with disease progression** **(****11****)**	**Mean number of seizures pre-treatment**	**Mean number of seizures post-treatment**	***P*****-value**
**ITT** (11 pts)	10.1 ± 11.8	5.6 ± 8.1	0.007
PP Only evaluable at 12 months (3 pts)	14.0 ± 13.9	7.0 ± 11.3	0.06
**Pts without disease progression** **(****18****)**	**Mean number of seizures pre-treatment**	**Mean number of seizures post-treatment**	***P*****-value**
**ITT** (25 pts)	8.6 ± 13.5	1.3 ± 1.8	0.01
PP Only evaluable at 12 months (19 pts)	10.1 ± 15.1	0.9 ± 1.1	0.01

At basal evaluation QOLIE 31-p was administered in 25 out of 36 patients; the remaining 11 did not perform the test because they were aphasics (5) and had scarce compliance (6). At final follow-up QOLIE 31-p was administered in 17 out of the 25 baseline that performed it at baseline: eight did not complete the questionnaire because five dropped out (four because of disease progression and one for side effects) and three had scarce compliance.

In these 17 patients, the comparison between baseline and final follow-up did not show any statistically significant difference in QOLIE global score (basal: 50.0 ± 20.6; final follow-up: 56.5 ± 17.9; *p* = 0.14), and values remained stable, within normal ranges. Although not statistically significant, all the sub-items (seizure worry, overall quality of life, emotional well-being, energy-fatigue, cognitive, medication effect, social function) had a tendency to improve after PER treatment, particularly social functioning (see Table 2D). In order to assess a possible influence of the oncological disease on QOLIE 31-p responses, we made a comparison between QOLIE 31-p mean scores in patients with disease progression vs. patients without disease progression at baseline and at final follow-up. Our results showed no significant difference between the two groups (QOLIE global score at baseline: 52.4 ± 15.3 in patients with disease progression and 50.6 ± 21.2 in patients without disease progression; *p* = 0.84; QOLIE global score at final follow up: 54.0 ± 13.8 in patients with disease progression; 56.8 ± 18.7 in patients without disease progression; *p* = 0.84).

**Table 2D:** Results in QOLIE 31-P test in 17 patients before and after 12 months of follow-up with PER.

**QOLIE 31-P**	**Basal**	**12 months f-u**	***P*-value**
	**(Mean ± SD)**	**(Mean ± SD)**	
Seizure worry	33.9.2 ±25.7	40.2 ± 23.6	0.36
Quality of life	58.0 ± 20.1	60.8 ± 17.6	0.48
Emotional well-being	55.4 ± 22.9	58.3 ± 22.3	0.53
Energy/fatigue	48.0 ± 24.9	48.6 ± 21.8	0.91
Cognitive	47.0 ± 28.0	56.9 ± 26.7	0.19
MEDS effect	45.5 ± 33.6	49.8 ± 28.1	0.56
Social functioning	53.9 ± 28.4	65.4 ± 22.8	0.08
QOLIE global score	50.0 ± 20.6	56.5 ± 17.9	0.14
QOLIE global score (*T*-score)	44.2 ± 13.3	46.4 ± 10.6	0.39

The AQ and BHS were only available for seven patients both at baseline and at the end of the study. Despite the low number of patients with complete data, aggression, and hopelessness measured by the tests did not seem to be worsened by PER (see Supplementary Table 3).

## Discussion

Epilepsy might be a difficult to treat condition in patients with a brain tumor. Despite several ASMs are available, the treatment often fails to control the seizures and is associated with significant side effects. Different antiseizure medications, including tiagabine, pregabalin, oxcarbazepine, levetiracetam, valproic acid, topiramate, zonisamide, lacosamide, and lamotrigine have been used to treat BTRE (19, 20). According to literature evidence, there is not a consensus suggesting any specific drug in patients with BTRE. Also, there are no studies linking the choice of a drug to a specific molecular marker, or to type, and location of the tumor. The available landscape of ASMs is limited by the possible side effects, that are known to be more frequent in patients with BTRE, and moreover by the enzyme inducers properties interfering with the anti-cancer medications. In fact carbamazepine, phenytoin, and phenobarbital should not be considered. Today, it is commonly accepted that the newer generation drugs should be considered as first choice and among them are levetiracetam, lamotrigine, and topiramate (20, 21). Valproic acid should also be considered (22).

To date there have been only four studies and one case report evaluating the efficacy and safety of PER in BRTE (12–16). The first report was a clinical case about a 48 years old man with a multiform glioblastoma who achieved a sustained seizure freedom on PER until his death (14). The other three are small group studies.

Dunn-Pirio et al. studied eight patients with glioma-related focal-onset epilepsy: three out of eight had self-reported seizure reduction and an additional three reported improved control. Of the six patients that benefitted from PER therapy, five had IDH1mutant gliomas (15).

Izumoto et al. (16) reported seizure control (more than 50% seizure reduction) in ten analyzed patients (100%) and 6 patients (60%) became seizure-free.

Vecht et al. studied 12 drug resistant patients with BTRE. They reported an objective seizure response in nine out of 12 patients (75%): 50%-seizure reduction in three, seizure-freedom in six. They also commented that the seizure response was obtained early following antitumor-directed therapy in gliomas which could be considered as a surrogate-marker of early tumor responses, advancing confirmation by MR imaging by 6 months or more (14).

Recently, in our retrospective study, we have obtained a high rate (81.8%) of responders and five seizure-free patients out of 11 patients suggesting that PER could have been a therapeutic option in BTRE (13).

The PERADET study is the largest and the only multicentric, prospective study with a 12 months follow up period. It demonstrates a statistically significant efficacy of PER at 12 months with a significant seizure reduction in both the ITT and the PP population. The responder rate at the end of the study was as high as 90.4% and there were a 33.3% patients being seizures free which lead to a 58.3% retention rate at 12 months. The responder rate dropped but still remained substantial when the whole ITT population was considered reaching a 66.6 and 25% of patients seizure free. Indeed this is higher than the 39% responder rate reported in the largest real-world data assessing people affected by treatment resistant focal epilepsy (23).

The efficacy, in terms of seizure reduction was confirmed for both types of seizures, namely focal and bilateral confirming the efficacy of PER in both focal onset and bilateral seizures (24). With regard to the grade, we observed a statistically significant mean seizure reduction in HGG patients while we could not demonstrate it for LGG patients (possibly due the low number of patients in both groups). In our population both groups, patients with tumor progression during follow-up (11) and patients without tumor progression (18) had a significant seizure reduction between baseline and final follow-up. Literature data indicate that a re-occurrence of seizure can indicate a tumor growth (18, 25). Our results indicate that in our patients PER maintains a good efficacy over time despite radiologically evidenced disease progression. Both patients with IDH1 mutated and patients with MGMT methylated seemed to better respond to PER treatment, in agreement with the data reported by Dunn-Pirio et al. (15). Unfortunately only few patients were able to perform molecular analysis; therefore these data must be interpreted with extreme caution.

PER was also well-tolerated; in fact, although one-third of the entire cohort suffered an adverse event, none of these was considered severe and only three patients dropped off because of tolerability issues. Furthermore, in this study tolerability was better than reported by Rohracher et al. (23) in the largest real world study on perampanel that detected a 69% of treatment related adverse events.

Regarding response to QOLIE 31-p, questionnaire's mean scores were in normal ranges at basal evaluation and remained stable at final follow-up, showing no significant differences. Literature data on BTRE populations indicate that patients treated with new generation ASMs as add-on therapy, show stability in QOLIE 31-P domains after 6 months of treatment, despite they achieve good seizure control. Our results on patients' perceived quality of life are in line with this evidence. However, our results could be also affected by the low number of patients who were able to perform the tests at 12 months' follow-up (17 out of 36). In addiction, although QOLIE 31-p is a test specifically designed to assess quality of life related to seizures disorders, we also know that quality of life in cancer patients is multifactorial as they may experience distress from the diagnosis, the effects of the disease, progression, treatment, and side effects (26). However, in our patients the statistical analysis showed no significant difference in QOLIE 31-p mean scores between patients with disease progression compared to patients without, suggesting that the oncological disease did not seem to influence QOLIE 31-p responses, as shown by previous literature evidences (27).

This study has some limitations. A subgroup analysis with regard to the histology and/or location of the tumor was not possible as the population was quite heterogeneous. Regarding the tumor grade, we could observe a significant mean seizure change in the HGG while we failed to demonstrate it in LGG. However, we believe that, given the small number of patients per each subgroup, this data need to be taken with caution and need further confirmation with a larger cohort. Also, the molecular features were not available for all patients. Furthermore, data regarding aggression and hopelessness, while being positive were limited to only seven patients and cannot be currently taken into account.

## Conclusion

PERADET is the largest BRTE population studied so far, allowing the confirmation of a good efficacy and tolerability of PER in BRTE that was already reported in smaller studies.

In the future, a broader collaborative study with a comprehensive characterization of the histology and molecular details of the tumor, could better clarify what type of patients/tumor might be more suitable for this treatment, nevertheless, per seems a promising therapeutic intervention for a substantial group of patients with BTRE.

## Data Availability Statement

All datasets generated for this study are included in the article/Supplementary Material.

## Ethics Statement

The studies involving human participants were reviewed and approved by Approved by the Ethical Committee (Prot. n° 0008872.25-06-2019; RS 919/17) of IRCCS IFO ‘Regina Elena' National Cancer Institute. The patients/participants provided their written informed consent to participate in this study.

## Consent for Publication

Written informed consent for publication of their clinical details and/or clinical images was obtained from the patient. A copy of the consent form is available for review by the Editor of this journal.

## Author Contributions

AC: study design, collection of the cases, writing of the manuscript, editing of the manuscript. AZ: collection of the cases. AM: collection of the cases. VV: collection of the cases. TK: collection of the cases. ER: study design and editing of the manuscript. AN, CS, VB, and EF: collection of the cases. SB: study design. DG: study design, statistical analysis. LB: collection of the cases. MM: study design, collection of the cases, writing of the manuscript, and editing of the manuscript. All authors contributed to the article and approved the submitted version.

## Conflict of Interest

AC has received speaker fees by Eisai. ER has received speaker fees and participated at advisory boards for Eisai and has received research fundings by GW Pharmaceuticals, Pfizer, Italian Ministry of Health (MoH) and the Italian Medicine Agency (AIFA). EF received speaker honoraria from EISAI and UCB. MM has received support for travel to congresses from EISAI srl; has participated in scientific advisory boards for EISAI; has participated in pharmaceutical industry-sponsored symposia for UCB Pharma; has received research grants from UCB Pharma. The remaining authors declare that the research was conducted in the absence of any commercial or financial relationships that could be construed as a potential conflict of interest.

## Abbreviations

AQ: aggression questionnaire
AEs: adverse events
ASMs: anti-seizure medications
BHS: Becks hopelessness scale
BRTE: brain tumor related epilepsy
1-IDH1: isocitrate dehydrogenase
ITT: intention to treat
MGMT: O6-Methylguanine-DNA-methyltransferase
PER: perampanel
PP: per protocol
QoL: quality of Life
QOLIE: quality of life in Epilepsy.
