# Supplementary material for: Perampanel Confirms to Be Effective and Well-Tolerated as an Add-On Treatment in Patients With Brain Tumor-Related Epilepsy (PERADET Study)
Source: Front Neurol. 2020 Jun 25;11:592. doi: 10.3389/fneur.2020.00592 (PMC7336340; doi:10.3389/fneur.2020.00592)
Supplement: Supplementary file 1 [file Data_Sheet_1.docx]

**Supplementary table 1: Adverse events related to dose regimen**

|  | | | | | |
| --- | --- | --- | --- | --- | --- |
|  | | | AE | | Total |
|  |  |  | YES | NO |  |
| DOSE_PER__mg_die | 2,0 | Count | 0 | 1 | 1 |
|  |  | % per_mg_die | 0,0% | 100,0% | 100,0% |
|  | 4,0 | Count | 3 | 4 | 7 |
|  |  | % per_mg_die | 42,9% | 57,1% | 100,0% |
|  | 6,0 | Count | 4 | 10 | 14 |
|  |  | % per_mg_die | 28,6% | 71,4% | 100,0% |
|  | 7,0 | Count | 1 | 0 | 1 |
|  |  | % per_mg_die | 100,0% | 0,0% | 100,0% |
|  | 8,0 | Count | 1 | 8 | 9 |
|  |  | % per_mg_die | 11,1% | 88,9% | 100,0% |
|  | 10,0 | Count | 2 | 1 | 3 |
|  |  | % per_mg_die | 66,7% | 33,3% | 100,0% |
|  | 12,0 | Count | 0 | 1 | 1 |
|  |  | % per_mg_die | 0,0% | 100,0% | 100,0% |
| Total | | Count | 11 | 25 | 36 |
|  |  | % per_mg_die | 30,6% | 69,4% | 100,0% |

| **Supplementary table 2: type of Adverse events related to dose regimen** | | | | | | | |
| --- | --- | --- | --- | --- | --- | --- | --- |
|  | | | AE | | | | Total |
|  |  |  | anxiety | aggressiveness | dizziness | fatigue |  |
| DOSE_PER__mg_die | 4,0 | Count | 1 | 0 | 1 | 1 | 3 |
|  |  | % per_mg_die | 33,3% | 0,0% | 33,3% | 33,3% | 100,0% |
|  | 6,0 | Count | 0 | 1 | 3 | 0 | 4 |
|  |  | % per_mg_die | 0,0% | 25,0% | 75,0% | 0,0% | 100,0% |
|  | 7,0 | Count | 1 | 0 | 0 | 0 | 1 |
|  |  | % per_mg_die | 100,0% | 0,0% | 0,0% | 0,0% | 100,0% |
|  | 8,0 | Count | 0 | 0 | 0 | 1 | 1 |
|  |  | % per_mg_die | 0,0% | 0,0% | 0,0% | 100,0% | 100,0% |
|  | 10,0 | Count | 0 | 1 | 1 | 0 | 2 |
|  |  | % per_mg_die | 0,0% | 50,0% | 50,0% | 0,0% | 100,0% |
| Total | | Count | 2 | 2 | 5 | 2 | 11 |
|  |  | % per_mg_die | 18,2% | 18,2% | 45,5% | 18,2% | 100,0% |

**Supplementary table 3: Aggressiveness and Helplessness scale evaluated for a subgroup of 7 patients**

| Test | Subscale | Baseline | 6 months | 12 months |
| --- | --- | --- | --- | --- |
| Aggression Questionnaire | Physical | 15,6 | 18,3 | 17,8 |
|  | Verbal | 14 | 16,6 | 15,5 |
|  | Anger | 18,2 | 19,3 | 19,5 |
|  | Hostility | 20,5 | 20,2 | 18 |
|  | Total | 68,3 | 74,6 | 71 |
| Beck Hopelessness Scale | Minimum (0-3) | 3/10 | 5/8 | 2/7 |
|  | Mild (4-8) | 5/10 | 0/08 | 2/7 |
|  | Moderate (9-14) | 0/10 | 2/8 | 2/7 |
|  | Severe (15-20) | 2/10 | 1/8 | 1/8 |
|  | Median score | 7,1 | 5,8 | 7,4 |
